# Odon: an ultra-fast viewer for spatial proteomics

**DOI:** 10.1093/bioinformatics/btag514

**Published:** 2026-07-11

**Authors:** Alexander Coulton, Nicholas McGranahan

**Affiliations:** Research Department of Oncology, UCL Cancer Institute, London WC1E 6DD, United Kingdom; Research Department of Oncology, UCL Cancer Institute, London WC1E 6DD, United Kingdom

## Abstract

**Motivation:**

Multiplexed spatial proteomics and spatial transcriptomics generate large, high-dimensional imaging datasets that are challenging to visualize efficiently, particularly at whole-slide and cohort scale. Visualization is an essential step for rapid detection of staining artefacts, such as protein aggregates or non-specific staining.

**Results:**

Here, we present Odon, a native Rust desktop viewer designed for rapid, interactive exploration of multiplex imaging data on a standard laptop. Odon is primarily built around the OME-Zarr imaging format, and supports annotations via GeoJSON and GeoParquet, with secondary support for SpatialData, Xenium containers, and TIFF. Data can be stored locally or streamed directly from HTTP or S3-compatible object storage using viewport-driven tile loading. Odon incorporates a highly optimized rendering engine designed for viewport-driven tile loading and GPU-based compositing. In scripted benchmarks using synthetic multiplex OME-Zarr datasets, Odon showed lower peak memory use, lower affine-derived zoom-step error, and faster warm-start image loading than napari and QuPath under the tested conditions. Its GPU-based compositing pipeline also enables smooth rendering and interaction with >1 000 000 segmented cells. Odon further supports integrated visual analytics, including live thresholding and cell selection, and a mosaic mode for simultaneous viewing of hundreds of regions of interest in cohort and tissue microarray studies. Together, these features establish Odon as a high-performance platform for scalable visualization of spatial proteomics data.

**Availability and implementation:**

Source code and compiled installers are available at https://github.com/alexcoulton/odon

## 1 Introduction

Spatial proteomics, a powerful method for the investigation of tissue architecture and tumour microenvironments (Wu *et al.* 2024, Xu *et al.* 2024), poses a unique data challenge. Multiplexed platforms such as Akoya PhenoCycler (Goltsev *et al.* 2018) and the Miltenyi Biotec MACSima (Kinkhabwala *et al.* 2022) can produce up to 100 high-resolution images of the same tissue section at subcellular resolution, often spanning entire slides, which, when scaled across a whole cohort can easily stretch into many terabytes of data. Visual inspection of this data remains a core step for both quality control and analysis. Image stitching errors, antibody aggregates, and suboptimal staining are all easily detected by eye. To accommodate this workflow and the sheer size of the data, many labs rely on using a powerful workstation with heavy technical specifications. Given that a single lab or institute will often only have one machine, this leads to a bottleneck in the analysis of the data: only one person can use the machine at a time, whereas a large lab group may have multiple simultaneous projects requiring the machine.

In this study, we leverage two design principles to solve this problem, creating an ultra-fast viewer for spatial proteomics, capable of rendering images of hundreds of samples simultaneously in the same viewer on standard consumer hardware. Firstly, our viewer is constructed using OME-Zarr (Moore *et al.* 2021) as a core file format, which is a next-generation, cloud-ready file format for biomedical imaging data, and has been demonstrated to outperform TIFF in terms of chunk retrieval time both locally and remotely (either via S3 or HTTP) (Moore *et al.* 2021).

Secondly, we employ the systems programming capabilities provided by Rust, a compiled language with strong benchmarked runtime performance (Pereira *et al.* 2017). This provides a strong foundation for efficient image rendering compared with viewers implemented in higher-level languages, including QuPath in Java (Bankhead *et al.* 2017) and napari in Python (Chiu, Clack, and the napari community 2022) while recognizing that overall performance also depends on the broader image loading, caching, and display pipeline. By pairing the lazy-loading capabilities of the OME-Zarr format with Rust’s memory efficiency and robust multi-threading, we have engineered a lightweight solution capable of rapidly rendering high-plex marker panels, whole-slide images, and tissue microarrays. This optimized architecture can lower the barrier to entry for spatial data visualization, allowing researchers to perform essential quality control and visual analyses on standard laptops rather than relying on specialized workstations. Odon derives its name from the dragonfly (Odonata), which is reported to exhibit ultra-fast sensitivity to flickering light (Ruck 1961).

## 2 Materials and methods

Benchmarking was performed on a 2024 MacBook Pro with an Apple M4 chip, 16 GB RAM, 1 TB SSD storage, and macOS Sequoia 15.5. Odon v0.1.5 was compared with QuPath version 0.7.0 and napari version 0.6.6 running under Python 3.11.13. Benchmarks used synthetic highly multiplexed OME-Zarr image pyramids generated at increasing scale, containing 10k, 50k, 100k, 500k, or 1 m simulated cells. Each dataset contained 24 synthetic multiplex channels and was stored as a multiscale OME-Zarr pyramid.

We performed four viewer benchmarks. First, peak memory usage was measured during a scripted zoom-in/zoom-out interaction using the 10k, 100k, and 1 m OME-Zarr datasets with one visible channel. Resident set size was sampled during each run and the peak value was recorded. Second, zoom consistency was assessed from screen recordings of a scripted 3 s zoom-in followed immediately by a 3 s zoom-out. For each adjacent video frame pair, an affine transform was estimated and converted into a frame-to-frame log-scale zoom step. The plotted metric is the 95th percentile absolute error between the observed affine-derived zoom step and the ideal scripted zoom step, where lower values indicate smoother zoom behaviour. QuPath runs in which level-0 tiles did not visibly resolve during the benchmark window were marked separately. Third, warm-start image load time was measured for 10k, 50k, 100k, 500k, and 1 m OME-Zarr datasets, with all channels visible. Warm start indicates that application startup time was excluded; timing began when image loading was initiated in an already open viewer and ended when the image was first visible in a usable state. Fourth, level-0 tile loading was measured after a rapid zoom into the 1 m-cell image, comparing OME-Zarr and OME-TIFF inputs for Odon and QuPath.

All benchmarks were performed with three replicate runs. Screen-recorded benchmarks were captured at 60 fps and manually timed in Adobe Premiere using frame-accurate timecodes. Further details on dataset generation, viewer automation, timing, affine-transform analysis, and benchmark scripts are provided in the Supplementary Methods, available as supplementary data at *Bioinformatics* online.

## 3 Results

**Figure 1:**
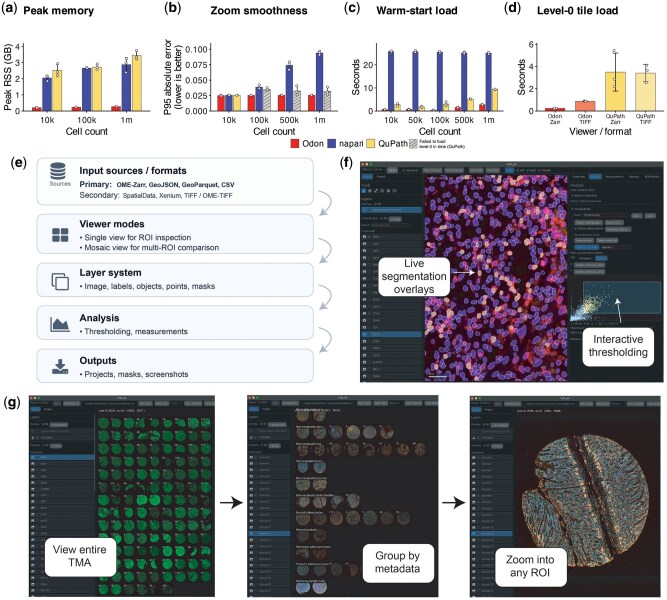
Overview of Odon workflow, benchmarking, and viewer modes. (a–d) Benchmark comparison of Odon, napari, and QuPath using synthetic OME-Zarr viewer workloads. Panels show peak resident memory during a scripted zoom-in/out interaction (a), P95 absolute affine zoom-step error during the same zoom benchmark, where lower is better and grey hatched QuPath bars indicate runs where level-0 tiles did not visibly resolve (b), warm-start time to first viewable image with all channels visible (c), and time to resolve level-0 viewport tiles after a rapid zoom into the 1 m-cell image for Odon and QuPath using OME-Zarr and OME-TIFF inputs (d). (e) Summary of Odon input formats, viewer modes, layer system, analysis tools, and outputs. (f) Single-view inspection with live segmentation overlays and interactive thresholding. (g) Mosaic mode for viewing, grouping, and zooming into many ROIs, such as tissue microarray cores. Icons adapted from Font Awesome Free by Fonticons, Inc., licensed under CC BY 4.0.

Odon is a native Rust desktop viewer built to handle the increasing scale of multiplex imaging and spatial omics data. The application primarily supports OME-Zarr, GeoJSON, and GeoParquet, with secondary support for SpatialData, Xenium containers, and TIFF (Fig. 1e). To reduce local storage bottlenecks, Odon can stream image data directly from HTTP or S3-compatible object storage using viewport-driven tile loading rather than full dataset downloads.

We benchmarked Odon against napari and QuPath using synthetic highly multiplexed OME-Zarr datasets containing 10k, 50k, 100k, 500k, or 1 m simulated cells. Across three replicate runs, Odon used less peak resident memory during scripted zoom-in/out interactions, showed lower affine-derived zoom-step error (i.e. smoother zooming) during screen-recorded zoom benchmarks, and achieved faster warm-start loading to a viewable image state than the comparator viewers under the tested conditions (Fig. 1a–c). In a separate rapid-zoom benchmark on the 1 m-cell dataset, Odon resolved level-0 viewport tiles faster than QuPath for both OME-Zarr and OME-TIFF inputs (Fig. 1d). QuPath runs in which level-0 (highest resolution) tiles did not visibly resolve during the 6-second benchmark window are marked separately in the figure. Separately, we confirmed that Odon can load segmentation objects from GeoParquet files and maintain responsive rendering and interaction with overlays containing >10 00 000 cell objects (see Supplementary data, available as supplementary data at *Bioinformatics* online).

The viewer facilitates granular exploration through a unified layer system supporting images, labels, and masks. Users can seamlessly overlay high-density segmentation maps and interrogate single-cell data in real time (Fig. 1f). Odon integrates analysis tools directly into the viewer, including object-property histograms and scatter plots that drive live, on-canvas thresholding and cell selection. Odon can also be controlled through a Model Context Protocol (MCP)-compatible automation interface, allowing external coding agents to open datasets, restore viewer state, apply annotations, and create biologically meaningful marker groups, for example grouping T-cell, B-cell, myeloid, or stromal markers for rapid inspection.

To address the needs of cohort-level studies and tissue microarrays (TMAs), Odon includes a dedicated mosaic mode that displays multiple regions of interest (ROIs) on a single canvas (Fig. 1g). This environment is driven by project workspaces or sample sheets, allowing users to automatically group, sort, and label hundreds of tissue cores based on associated metadata. Users can evaluate marker expression across an entire cohort simultaneously and immediately zoom into any specific ROI for high-resolution inspection.

Whilst Odon is designed to be a fast viewer, it is not designed to replace the full feature set of more extensive image analysis platforms (like QuPath and napari). For example, it does not provide a means for running cell segmentation algorithms and is limited to a smaller set of imaging file formats. The suggested workflow is to run these algorithms on a computing cluster and use Odon to visualize the results.

## Supplementary Material

btag514_Supplementary_Data

## Data Availability

The benchmark datasets used for the revised viewer comparisons were generated synthetically as OME-Zarr image pyramids to provide reproducible workloads at defined cell counts. These OME-Zarrs, as well as accompanying segmentation data, are available from the BioImage Archive (https://www.ebi.ac.uk/biostudies/bioimages/studies/S-BIAD3559).

## Code availability

Odon is available at https://github.com/alexcoulton/odon as well as from Zenodo (Odon v0.1.5) https://doi.org/10.5281/zenodo.20646059. The scripts used to generate the synthetic datasets, run the benchmarks, and analyse the results are available both on GitHub (https://github.com/alexcoulton/odon_benchmark/) and Zenodo (https://doi.org/10.5281/zenodo.20644561).
