# Supplementary material for: Odon: an ultra-fast viewer for spatial proteomics
Source: Bioinformatics. 2026 Jul 11;42(7):btag514. doi: 10.1093/bioinformatics/btag514 (PMC13394687; doi:10.1093/bioinformatics/btag514)
Supplement: btag514_Supplementary_Data [file btag514_supplementary_data.pdf]

Odon: An ultra-fast viewer for spatial proteomics - supplementary methods

2026-06-11

1 Purpose

This document describes the benchmark methodology used to compare Odon with napari and QuPath for interactive visualization of large, highly multiplexed spatial proteomics images. It provides the detailed benchmark design, measurement definitions, formulas, software dependencies, scripts, and data flow used to generate the publication benchmark figure.

The benchmark suite measures four complementary properties of viewer behaviour:

- peak memory use during a scripted zoom-in/zoom-out interaction;
- affine-derived zoom-step error during a screen-recorded scripted zoom-in/zoom-out interaction;
- warm-start image load time after the viewer is already open;
- time to resolve level-0 viewport tiles after a rapid zoom into a 1m-cell image.

2 General Benchmarking Principles

The benchmarks are intended to evaluate a focused visualization workload: opening, displaying, and navigating large multiplexed image pyramids. They are not intended to compare the full feature sets of the three applications.

The same dataset conditions and high-level interaction pattern were used across viewers wherever possible. Viewer-specific scripting was required because Odon, napari, and QuPath expose different automation interfaces:

- Odon was controlled through an MCP interface that can open images, set channel visibility, set contrast limits, set the camera, hide side panels, and disable smooth interpolation.
- napari was controlled through Python scripts that construct a napari viewer, load the image, configure visible layers, and animate the camera.
- QuPath was controlled by Groovy scripts run inside QuPath. For QuPath, scripts were placed on the clipboard from shell commands and then executed in the QuPath script editor.

All final quantitative benchmarks use three replicate measurements. Manual screen-recording measurements used Adobe Premiere timecodes in `MM:SS:FF` format at 60 frames per second. A timecode such as `00:05:26` therefore means 5 seconds plus 26/60 seconds, not 5 minutes and 26 seconds.

Final scripts and generated helper files are preserved in `benchmark_scripts/`. Exploratory scripts that were tested during method development but not used in the final presented benchmarks are intentionally excluded from the distributed benchmark bundle.

3 Hardware and Software

Benchmarking was performed on a 2024 MacBook Pro with an Apple M4 chip, 16 GB RAM, 1 TB SSD storage, and macOS Sequoia 15.5.

Viewer versions:

- Odon: v0.1.5.
- napari: version 0.6.6 running under Python 3.11.13.
- QuPath: version 0.7.0 with the Bio-Formats extension for OME-Zarr and OME-TIFF reading.

External command-line tools and applications:

- OBS was used for screen recording at 60 fps.
- Adobe Premiere was used to manually identify visible timing intervals in the screen recordings.
- `ffmpeg` and `ffprobe` were used to decode video frames, trim clips, and inspect clip duration/frame-rate metadata.
- macOS `ps` was used for process memory sampling.
- macOS `open`, `pkill`, `nohup`, and `pbcopy` were used in the shell scripts to launch viewers, stop viewers, run background processes, and copy QuPath Groovy scripts.
- XeLaTeX was used to compile this supplementary methods PDF from the generated LaTeX source.

Python dependencies for the benchmark analysis scripts are recorded in `pyproject.toml` and the accompanying lock file. The main packages used by the final benchmark scripts are:

- `numpy`: frame arrays and image processing data structures.
- `pandas`: replicate-level CSV parsing, timecode-derived timing tables, and grouped summary statistics.
- `opencv-python-headless`: ECC affine transform estimation and image writing for affine-transform previews.
- Python standard library modules including `argparse`, `csv`, `json`, `statistics`, `subprocess`, `pathlib`, `time`, and `signal`.

R dependencies for the manuscript-style summary figure are:

- `readr`: reading and writing CSV files.
- `dplyr`: table transformation and summarisation.
- `ggplot2`: plotting benchmark panels.
- `patchwork`: combining panels into a compact multi-panel figure.
- `scales`: axis and label formatting.

4 Dataset Conditions

The benchmark datasets are synthetic highly multiplexed OME-Zarr image pyramids generated at multiple cell-count scales:

- 10k cells;
- 50k cells;
- 100k cells;
- 500k cells;
- 1m cells.

The final benchmark panels use these subsets:

- Peak memory: 10k, 100k, and 1m OME-Zarr, 1 visible channel.

- Zoom-step error: 10k, 100k, 500k, and 1m OME-Zarr, 1 visible channel.
- Warm-start load time: 10k, 50k, 100k, 500k, and 1m OME-Zarr, all channels visible.
- Level-0 tile-load time: 1m OME-Zarr and matching 1m OME-TIFF, Odon and QuPath only.

The benchmark shell scripts define local dataset paths. These paths are retained for transparency and must be edited when reproducing the benchmarks on another machine.

The OME-Zarr datasets used for the viewer benchmarks are deposited in the BioImage Archive under accession `S-BIAD3559`: https://www.ebi.ac.uk/biostudies/bioimages/studies/S-BIAD3559.

5 Synthetic OME-Zarr Generation

5.1 Source Repository

The benchmark OME-Zarr datasets were generated with a separate synthetic multiplex immunofluorescence data-generation repository. The relevant generator scripts are copied into the distributed benchmark bundle, so the methods below refer to bundled script paths rather than machine-specific local paths.

The source repository contains the original Synplex MATLAB simulator and additional Python scripts for generating larger OME-Zarr fixtures. Synplex is a simulator for multiplex immunofluorescence images, but the large benchmark OME-Zarrs used here were generated with the standalone Python generator rather than by running the MATLAB Synplex workflow directly.

The relevant generator scripts are archived in the synthetic generation folder of the bundled benchmark scripts:

- `00_01_synthetic_omezarr_generation_file_descriptions.md`: folder-local inventory describing the synthetic data-generation files.
- `01_generate_synthetic_mif.py`: core synthetic MIF rendering, channel definitions, cell morphology model, intensity model, OME-Zarr writing, pyramids, labels, object tables, and preview generation.
- `02_generate_synthetic_mif_packed_parallel.py`: parallel packed-cell generator used for the final large benchmark OME-Zarrs. This wraps the core renderer but adds globally non-overlapping object placement and parallel chunk rendering.
- `generate_synthetic_mif.py`: import-compatible copy of the core renderer used by the packed parallel generator.

5.2 Dataset Sizes and Metadata

The final OME-Zarr benchmark datasets were square 24-channel image pyramids with 5 pyramid levels, 512 x 512 pixel chunks, and `t,c,z,y,x` axes. The generated image arrays used `uint16`; cell and nucleus label arrays used `uint32`.

The generated datasets were as follows. The size columns report the exact byte size of each generated compressed image OME-Zarr archive, named `image.ome.zarr.zip`, with decimal GB shown for readability. The OME-Zarr archives are available from the BioImage Archive deposition listed above.

Dataset	Level-0 pixels	Target cells	Placed cells	Seed	Archive bytes	Archive GB
10k	4096 x 4096	10,000	10,000	80	887,503,808	0.888
50k	8192 x 8192	50,000	49,782	75	3,501,505,790	3.502
100k	11776 x 11776	100,000	99,996	78	7,164,424,452	7.164
500k	26112 x 26112	500,000	500,000	77	34,602,540,727	34.603
1m	36864 x 36864	1,000,000	1,000,000	76	68,525,054,076	68.525

The 50k dataset contains slightly fewer placed cells than requested because the packed non-overlap constraint prevented all requested objects from being placed within the maximum placement-attempt budget.

5.3 Channel Model

Each OME-Zarr image contains 24 synthetic multiplex channels:

`DAPI`, `HistoneH3`, `Ki67`, `FOXP3`, `PanCK`, `EPCAM`, `NaKATPase`, `PDL1`, `CD45`, `CD3`, `CD8`, `PD1`, `CD68`, `CD163`, `Vimentin`, `SMA`, `CollagenI`, `Fibronectin`, `CD31`, `Laminin`, `Mito`, `GranzymeB`, `Autofluorescence`, and `BackgroundTexture`.

Each channel has an assigned display colour and a localization class:

- nuclear channels are rendered from nucleus labels;
- membrane channels are rendered from membrane labels;
- cytoplasm channels are rendered from cell labels excluding nucleus pixels;
- background channels are rendered from low-frequency stromal/background fields rather than object labels.

The OME-Zarr metadata includes OMERO-style channel metadata. Display windows were initialized with min 0, max 65535, start 0, and end 52000.

5.4 Cell Phenotypes

Synthetic cells were assigned one of five broad phenotypes:

- `tumor`;
- `immune_t`;
- `immune_myeloid`;
- `stroma`;
- `proliferative`.

Phenotype assignment was density-dependent. In the packed parallel generator, phenotype scores were calculated as:

$$\text{tumor} = 1.15\,\text{density}$$

$$\text{immune}_t = 0.45 + 0.45(1 - \text{density})$$

$$\text{immune}_{myeloid} = 0.25 + 0.25(1 - \text{density})$$

$$\text{stroma} = 0.55$$

$$\text{proliferative} = 0.18\,\text{density}$$

Scores were clipped to a minimum of 0.001, normalized to sum to 1, and sampled with a NumPy random generator.

5.5 Spatial Density Field

Cell placement was guided by a deterministic synthetic tissue-density field. Coordinates were normalized to the image dimensions. The field combined:

- a broad central radial component;
- a low-density notch;
- sinusoidal wave terms depending on normalized position and seed;

- a tissue mask threshold.

The density value was clipped to 0-1 and set to zero outside tissue:

$$\text{density} = \text{clip}(\text{radial} - 0.45\,\text{notch} + \text{waves}, 0, 1)$$

$$\text{tissue} = \text{density} > 0.16$$

$$\text{density} = \begin{cases} \text{density}, & \text{if tissue} \\ 0, & \text{otherwise} \end{cases}$$

This creates spatially heterogeneous regions so the benchmark images are not uniform random point fields.

5.6 Packed Cell Placement

Cells were placed in 1024 x 1024 pixel placement tiles. Tile-level target counts were assigned in proportion to density-weighted tile area. If an exact target object count was provided, the target count was distributed across tiles by:

1. calculating each tile's density-weighted area;
2. assigning a raw fractional target count proportional to that weight;
3. taking the floor of each raw target;
4. distributing remaining cells to tiles with the largest fractional remainders.

Within each tile, candidate cell centers were sampled with a 16 pixel border margin. Candidate acceptance required:

- non-zero density at the sampled location;
- a random density gate passing with probability up to `min(1, 0.30 + density)`;
- no overlap with nearby previously accepted cells.

The non-overlap criterion was:

$$d(\text{candidate}, \text{existing}) \geq 1.20(r_{\text{candidate}} + r_{\text{existing}})$$

A spatial hash was used to avoid comparing every candidate with every existing cell.

5.7 Cell Morphology

Each accepted cell was represented as an ellipse with a nucleus and membrane ring. The generator stored:

- center coordinates `x`, `y`;
- cell radius;
- nucleus radius;
- eccentricity;
- orientation angle;
- phenotype.

For packed datasets, the base cell radius was sampled from a normal distribution with mean 7.0 px and standard deviation 1.3 px, with phenotype-specific shifts:

- `immune_t`: -0.8 px;

- `immune_myeloid`: +0.1 px;
- `stroma`: +0.5 px;
- `tumor`: +0.9 px;
- `proliferative`: +0.7 px.

The resulting radius was clipped to 4.0-13.0 px. The nucleus radius was sampled as a fraction of the cell radius:

$$r_{\text{nucleus}} = r_{\text{cell}}\,\text{clip}(\mathcal{N}(0.52, 0.06), 0.38, 0.67)$$

Eccentricity was sampled as:

$$\text{eccentricity} = \text{clip}(\mathcal{N}(1.0, 0.08), 0.82, 1.18)$$

The orientation angle was sampled uniformly between 0 and pi.

5.8 Rasterization

For each render chunk, the generator found cells overlapping the chunk plus a halo margin. Each cell was rasterized as an oriented ellipse. The membrane mask was defined as pixels inside the cell ellipse with normalized radius at least 0.62. The nucleus was a smaller ellipse based on the sampled nucleus radius.

The generator wrote three level-0 arrays:

- multiplex image intensity: `image.ome.zarr`;
- cell instance labels: `labels/cells.ome.zarr`;
- nucleus instance labels: `labels/nuclei.ome.zarr`.

Image chunks were rendered in parallel. Each render task used a 16 pixel halo around the output chunk and queried cells within an additional 32 pixel margin to avoid edge artefacts at chunk boundaries.

5.9 Marker Expression and Intensity Rendering

Marker expression depended on both channel and phenotype. For example:

- tumor cells had high expression for `PanCK`, `EPCAM`, `NaKATPase`, `PDL1`, and `Mito`;
- T immune cells had high expression for `CD45`, `CD3`, `CD8`, `PD1`, and `GranzymeB`;
- myeloid immune cells had high expression for `CD45`, `CD68`, `CD163`, and `PDL1`;
- stromal cells had high expression for `Vimentin`, `SMA`, `CollagenI`, `Fibronectin`, and `Laminin`;
- proliferative cells had high expression for `PanCK`, `EPCAM`, `Ki67`, and `Mito`;
- `DAPI` and `HistoneH3` were high across phenotypes.

For each cell/channel pair, expression was calculated as:

$$\text{expression} = \text{clip}(\text{base}_{expression}\,\text{lognormal}(\mu = -0.03, \sigma = 0.38), 0, 1.25)$$

The random variation was deterministic for a given seed, cell ID, and channel name. Object-localized channel intensity was added as:

$$\text{expression} \times 46000$$

Background and illumination terms were added to every channel:

$$\text{illumination}\,[1200 + 1100((\text{channel}_{index} \text{ mod } 7) + 1)]$$

$$\text{density}\,[900 + 220(\text{channel}_{index} \text{ mod } 5)]$$

For background-localized channels, an additional stromal background term was added:

$$(0.15 + 0.85\,\text{stroma})\,\text{phenotypeExpression}(\text{channel}, \text{stroma}) \times 36000$$

Channels were Gaussian blurred according to localization:

- nuclear: sigma 0.55;
- membrane: sigma 0.95;
- cytoplasm/background: sigma 1.25.

Deterministic sinusoidal pseudo-noise was then added with amplitude:

$$650 + 0.015\,\text{max}(\text{image}, 1)$$

Final intensities were clipped to 0-65535 and stored as `uint16`.

5.10 Pyramid Generation

The generated OME-Zarrs use 5 multiscale levels with OME-NGFF 0.4 metadata. Level shapes were generated by repeated two-fold downsampling with ceiling division. Image pyramid levels were built by 2 x 2 mean downsampling. Label pyramid levels were built by nearest-neighbour subsampling to preserve integer instance labels.

All arrays used Zlib compression at level 1. Chunk shape was:

$$(t = 1,\ c = 1,\ z = 1,\ y \leq 512,\ x \leq 512)$$

Coordinate transformations in the OME-Zarr metadata encode scale factors of 1, 2, 4, 8, and 16 for the y and x axes across the five levels.

5.11 Object and Preview Outputs

For each generated dataset, the script wrote:

- `objects/cells.parquet`: GeoParquet cell polygons and scalar properties, compressed with zstd;
- `objects/cells.geojson`: GeoJSON cell polygons and scalar properties;
- `metadata.json`: generation settings, dimensions, placed object count, channel metadata, phenotype names, and sample cells;
- `preview.png`: RGB preview using PanCK, CD3, and DAPI.

Cell polygons were generated from the same oriented ellipse parameters used for rasterization. Each polygon used 20 points and was closed by repeating the first coordinate.

6 Script Bundle Layout

The publication script bundle is in `benchmark_scripts/`. The manifest `script_manifest.csv` records the original source path, bundled path, benchmark folder, role, and file size for each copied script asset.

The bundle is organized as follows:

- `00_environment/`: repository context and Python dependency provenance.
- `01_synthetic_omezarr_generation/`: synthetic multiplex image and OME-Zarr generation scripts.
- `02_peak_memory/`: peak RSS memory benchmark command sequence, viewer automation scripts, QuPath loader/-zoom scripts, and memory analysis scripts.
- `03_zoom_step_error/`: focused scripted zoom-in/out command reference, viewer automation scripts, QuPath zoom scripts, video trimming, affine zoom-step analysis, affine visualisation, composite-video generation, and shared helpers.
- `04_warm_start_load/`: warm-start load command sequence, screen marker, Odon/napari load drivers, QuPath warm-load script generator, generated QuPath warm-load scripts, and timing analysis.
- `05_level0_tile_load/`: focused rapid zoom-in command reference, Odon driver, QuPath fast zoom-in scripts, and level-0 tile-load timing analysis.
- `06_publication_outputs/`: scripts for curated publication tables, manuscript figures, supplementary media, script-bundle generation, and supplementary PDF generation.

Each benchmark folder contains a `00_`-prefixed Markdown inventory file describing every file in that folder. These folder-local inventories are intended as the most convenient human-readable index; `script_manifest.csv` is the machine-readable provenance table for copied script assets. Numbered files are workflow steps or command references; unnumbered Python files are helper modules retained under import-compatible names.

7 Benchmark 1: Peak Memory During Scripted Zoom

7.1 Aim

This benchmark measures process memory use while each viewer performs a standardized interactive task: zooming in for 3 seconds and then immediately zooming out for 3 seconds. The plotted metric is peak resident set size (RSS), in GB.

7.2 Viewer Setup

The final memory benchmark uses 1 visible channel for each viewer and the 10k, 100k, and 1m OME-Zarr datasets. Odon and QuPath were launched fresh where possible. napari used a warm-start trigger workflow: napari loaded the image first, wrote a ready file, and waited for a trigger file before starting the zoom. This allowed memory sampling to begin before the zoom animation.

The zoom animation used:

- zoom factor: 100;
- zoom-in duration: 3.0 s;
- zoom-out duration: 3.0 s;
- animation update target: 100 updates/s in the scripting layer;
- one visible channel.

Odon was run with side panels hidden and smooth interpolation disabled for the scripted zoom. napari was run with sidebars hidden for the scripted zoom. QuPath used a loader Groovy script followed by a zoom-only Groovy script for the warm-start memory workflow.

7.3 Memory Sampling

Memory was sampled with `monitor_memory.py` at 0.1 s intervals using macOS `ps`:

```
ps -axo pid=,ppid=,rss=,vsz=,command=
```

For each sample, the script matched one or more root processes using executable name or command-line patterns. If child-process inclusion was enabled, descendant processes were identified through parent PID relationships and included in the same sample. For a sample `t`, total RSS was calculated as:

$$\text{RSS}_{MB}(t) = \frac{\sum_{p \in P} \text{RSS}_{KB}(p,t)}{1024}$$

Virtual memory size was calculated analogously:

$$\text{VMS}_{MB}(t) = \frac{\sum_{p \in P} \text{VSZ}_{KB}(p,t)}{1024}$$

The script also recorded the maximum single-process RSS and VMS at each sample, the matched process count, matched PIDs, elapsed time, and process names.

7.4 Memory Summary Metrics

Only samples with at least one matched process were used for the memory summary. The first matched sample was treated as the baseline. Let `R(t)` be total RSS in MB for matched samples. The primary plotted metric was:

$$\text{peakRSS}_{MB} = \max_t R(t)$$

The value plotted in the manuscript figure is:

$$\text{peakRSS}_{GB} = \frac{\text{peakRSS}_{MB}}{1024}$$

Additional summary fields written by the scripts include:

- `baseline_rss_mb`: total RSS in the first matched sample;
- `delta_peak_rss_mb=peak_rss_mb-baseline_rss_mb`;
- `final_rss_mb`: total RSS in the last matched sample;
- `retained_delta_rss_mb=final_rss_mb-baseline_rss_mb`;
- `mean_rss_mb = mean_t R(t);`
- equivalent VMS fields.

Replicate summaries were generated with `analyze_memory_replicates.py`, which writes long-form replicate CSVs and grouped summary CSVs using `pandas`. The publication figure uses mean peak RSS across three replicates, with individual replicate points overlaid.

7.5 Relevant Scripts and Outputs

All memory benchmark scripts are archived under `benchmark_scripts/02_peak_memory/`:

- `00_02_peak_memory_file_descriptions.md`: folder-local inventory describing the memory benchmark files.
- `00_run_memory.sh`: top-level peak RSS memory benchmark command sequence.
- `01_monitor_memory.py`: process RSS memory sampler.
- `02_odon_scriptable_zoom.py`: Odon scripted zoom driver used by the memory benchmark.
- `03_napari_warm_scriptable_zoom.py`: napari warm-start zoom driver used by the memory benchmark.
- `04_01_qupath_load_10k_channels_1.groovy`: QuPath 10k 1-channel image loader for memory benchmarking.
- `04_02_qupath_load_100k_channels_1.groovy`: QuPath 100k 1-channel image loader for memory benchmarking.

- `04_03_qupath_load_1m_channels_1.groovy`: QuPath 1m 1-channel image loader for memory benchmarking.
- `05_01_qupath_zoom_only_10k_channels_1.groovy`: QuPath 10k warm-start zoom-only memory benchmark script.
- `05_02_qupath_zoom_only_100k_channels_1.groovy`: QuPath 100k warm-start zoom-only memory benchmark script.
- `05_03_qupath_zoom_only_1m_channels_1.groovy`: QuPath 1m warm-start zoom-only memory benchmark script.
- `06_summarize_memory.py`: memory summary table helper.
- `07_analyze_memory_replicates.py`: replicate-level peak RSS memory analysis.
- `napari_scriptable_zoom.py`: import-compatible napari helper used by the warm-start memory driver.

Main outputs:

- `data/raw_replicates/memory_peak_rss_1ch_replicates.csv`
- `data/memory_peak_rss_1ch_summary.csv`

8 Benchmark 2: Zoom-Step Error

8.1 Aim

This benchmark measures how consistently each viewer changes apparent zoom from frame to frame during a scripted zoom-in/zoom-out sequence. It uses screen-recorded video rather than viewer-internal timing, so the metric reflects visible output.

The final benchmark uses the 10k, 100k, 500k, and 1m OME-Zarr datasets with 1 visible channel. Each run consisted of a 3 s zoom-in followed immediately by a 3 s zoom-out. The animation factor was 100 and the scripting target was 100 updates/s.

8.2 Recording and Manual Intervals

The scripted runs were recorded with OBS at 60 fps. The raw capture was reviewed in Adobe Premiere and the visible start and end timecodes for each run were recorded as `MM:SS:FF`.

The timecode parser converts a Premiere timecode to frame index as:

$$\text{frameIndex} = ((\text{minutes} \times 60) + \text{seconds}) \times \text{fps} + \text{frames}$$

At 60 fps, conversion to seconds is:

$$t_{\text{seconds}} = 60\,\text{minutes} + \text{seconds} + \frac{\text{frames}}{60}$$

8.3 Refining the Active Zoom Window

Manual intervals can include still frames before the zoom begins or after it ends. These buffer frames were removed with `refine_manual_zoom_cuts.py`.

For each manually selected interval, frames were decoded with `ffmpeg`, downsampled to 320 x 180 grayscale, and adjacent-frame activity was calculated as normalized absolute pixel difference:

$$\text{activity}_i = \frac{\sum_p |F_{i+1}(p) - F_i(p)|}{\text{width} \times \text{height} \times 255}$$

The activity trace was smoothed with a moving average of radius 2 frames. An adaptive activity threshold was then calculated from the smoothed scores:

$$\text{low} = P_{10}(\text{activity})$$

$$\text{high} = P_{90}(\text{activity})$$

$$\text{median} = \text{median}(\text{activity})$$

$$\text{MAD} = \text{median}(|\text{activity} - \text{median}|)$$

$$\text{relativeThreshold} = \text{low} + 0.20(\text{high} - \text{low})$$

$$\text{robustThreshold} = \text{median} + 2\,\text{MAD}$$

$$\text{threshold} = \text{max}(0.001, \text{min}(\text{relativeThreshold}, \text{robustThreshold}))$$

The active range starts at the first 4-frame window where at least 3 frames are above threshold. It ends at the last 4-frame window satisfying the same condition. Two frames of padding were added at both ends. Trimmed clips were written with `ffmpeg` using H.264 (`libx264`), constant frame rate 60 fps, CRF 12, and `yuv420p` pixel format.

8.4 Affine Transform Estimation

The final zoom-step error metric uses affine transform estimation between adjacent frames. The trimmed clips were decoded with `ffmpeg`, downsampled to 640 x 360, converted to grayscale, and passed to OpenCV.

For adjacent frames `I_i` and `I_{i+1}`, `cv2.findTransformECC` estimated a 2 x 3 affine transform:

$$A = \begin{bmatrix} a & b & t_x \\ c & d & t_y \end{bmatrix}$$

The OpenCV mode was `cv2.MOTION_AFFINE`. The transform was initialized as the identity matrix. The ECC optimizer used:

- maximum iterations: 50;
- epsilon: 1e-5;
- Gaussian blur kernel: 5;
- minimum accepted ECC value: 0.05.

Frames were converted to `float32`, scaled to 0-1, and Gaussian blurred before ECC fitting. If ECC fitting failed, produced a non-finite value, or had ECC below 0.05, the frame pair was counted as a transform failure.

8.5 Frame-to-Frame Zoom Step

The affine matrix contains translation, rotation, shear, and scale. The apparent scale was estimated from the linear 2 x 2 component:

$$s_x = \sqrt{a^2 + c^2}$$

$$s_y = \sqrt{b^2 + d^2}$$

$$s = \frac{s_x + s_y}{2}$$

The frame-to-frame zoom step was defined as the absolute log scale change:

$$z_i = |\log(s_i)|$$

Using the log scale makes equal multiplicative zoom changes comparable regardless of whether the sequence is zooming in or zooming out. The absolute value is used because the plotted benchmark focuses on the magnitude and consistency of each frame-to-frame zoom step rather than the direction of the scripted phase.

8.6 Ideal Zoom Step

The scripted benchmark used a 100x zoom-in over 3 seconds followed by a 100x zoom-out over 3 seconds. The screen recordings were captured at 60 fps. Under a constant multiplicative zoom schedule, the ideal absolute log zoom step is:

$$z_{\text{ideal}} = \frac{\log(100)}{3 \times 60}$$

which evaluates to:

$$z_{\text{ideal}} = 0.0255842788$$

The ideal signed trace is positive during zoom-in and negative during zoom-out. For the absolute-error plot, only the magnitude of the ideal step is used.

8.7 P95 Absolute Zoom-Step Error

For each valid adjacent frame pair, the absolute error was:

$$e_i = |z_i - z_{\text{ideal}}|$$

The plotted metric was the 95th percentile of these frame-to-frame errors:

$$P95_{\text{abs}} = P_{95}(e_i)$$

This reports the upper-tail residual between the measured affine-derived zoom step and the ideal scripted zoom step. Lower values indicate more consistent frame-to-frame zoom. A value of zero would mean that at least 95% of measured frame pairs exactly matched the ideal log zoom step; this is a mathematical reference point rather than an expected value for real screen recordings.

The 95th percentile was used instead of the maximum because a maximum is highly sensitive to a single codec artefact, isolated frame, or affine-registration outlier. It was used instead of the mean because the mean can hide rare jumps or stalls that are visually apparent. Unlike the previous internal composite score, this metric does not use hand-chosen weights or jank/stall/spike thresholds.

8.8 Transform Failures

Transform failure rate was recorded separately:

$$\text{transformFailureRate} = \frac{\text{failureCount}}{\max(1, \text{validTransformCount} + \text{failureCount})}$$

Failed affine fits were not included in the P95 absolute-error calculation. They remain available in the replicate data and in the trace diagnostic plots as gaps or marked points. This keeps registration failure separate from measured zoom-step irregularity.

8.9 Cumulative Zoom Trajectory Diagnostic

The analysis also produced diagnostic cumulative zoom traces. Because the scripted phase is known, the unsigned zoom-step magnitude can be assigned a direction:

$$z_i^{signed} = +z_i \quad \text{during zoom-in}$$

$$z_i^{signed} = -z_i \quad \text{during zoom-out}$$

The observed cumulative trajectory is:

$$L_k = \sum_{i \le k} z_i^{signed}$$

and the corresponding ideal trajectory is:

$$L_k^{ideal} = \sum_{i \le k} z_i^{ideal,signed}$$

The replicate summaries include a secondary cumulative trajectory residual:

$$\text{RMSE}_L = \sqrt{\text{mean}((L_k - L_k^{ideal})^2)}$$

This diagnostic was used for method inspection but was not the manuscript panel metric. The cumulative plot is useful because stalls appear as flat segments, jumps appear as sudden steps, and lag appears as the observed curve falling behind the ideal ramp.

8.10 QuPath Level-0 Tile Resolution Flag

For QuPath 100k, 500k, and 1m runs, the viewer did not visibly resolve level-0 tiles during the benchmark window. These conditions are shown as grey hatched bars in the final figure. The flag is separate from the P95 affine zoom-step error because it captures high-resolution tile-resolution failure rather than motion residual alone.

8.11 Relevant Scripts and Outputs

All zoom-step error benchmark scripts are archived under `benchmark_scripts/03_zoom_step_error/`:

- `00_03_zoom_step_error_file_descriptions.md`: folder-local inventory describing the zoom-step error benchmark files.
- `00_run_zoom_step_error.sh`: focused command reference for the final 1-channel scripted zoom-in/out benchmark runs.
- `01_napari_scriptable_zoom.py`: napari scripted zoom-in/out driver.
- `02_odon_scriptable_zoom.py`: Odon scripted zoom-in/out driver.
- `03_01_qupath_scriptable_zoom_10k_channels_1.groovy`: QuPath 10k 1-channel scripted zoom-in/out benchmark.
- `03_02_qupath_scriptable_zoom_100k_channels_1.groovy`: QuPath 100k 1-channel scripted zoom-in/out benchmark.

- 03_03_qupath_scriptable_zoom_500k_channels_1.groovy: QuPath 500k 1-channel scripted zoom-in/out benchmark.
- 03_04_qupath_scriptable_zoom_1m_channels_1.groovy: QuPath 1m 1-channel scripted zoom-in/out benchmark.
- 04_cut_zoom_intervals.py: zoom interval clipping helper.
- 05_refine_manual_zoom_cuts.py: manual Premiere timecode refinement and clip trimming helper.
- 06_analyze_zoom_affine_trace.py: final affine-transform P95 zoom-step error analysis.
- 07_visualize_affine_transforms.py: affine-transform preview image generator.
- 08_make_viewer_composites.py: side-by-side composite clip generator.
- 09_plot_affine_zoom_trace.R: affine zoom-step trace and residual plot generator.
- affine_zoom_common.py: shared affine frame-decoding and ECC helper functions.
- zoom_trace_common.py: shared manifest, ffprobe, and percentile helpers for zoom trace analysis.

Main outputs:

- data/raw_replicates/zoom_step_error_1ch_affine_replicates.csv
- data/zoom_step_error_1ch_affine_summary.csv
- affine transform preview images in supplementary/affine_transform_examples/
- side-by-side clips in supplementary/composite_clips/

9 Benchmark 3: Warm-Start Load Time

9.1 Aim

Warm-start load time measures how long an already-open viewer takes to display a newly requested image in a usable state. This separates image-load responsiveness from application startup time.

9.2 Procedure

Each viewer was opened before the measured load began. The load was then triggered while the screen was being recorded. All channels were visible. Viewer default display behaviour was retained where possible. Odon used a visible max contrast scale of 0.9 for loaded channels to avoid an extremely dim initial view, and smooth interpolation was disabled.

The benchmark used the 10k, 50k, 100k, 500k, and 1m OME-Zarr datasets. Each viewer/dataset condition had three replicate measurements.

9.3 Visible Start Marker

A small screen marker was used to define the load start in the screen recording. The marker state was controlled by a marker file. Immediately before a load request, the scripts changed the marker so the start event was visible on screen even if the viewer covered the terminal. The measured start frame was the visible marker change corresponding to the load trigger.

9.4 Viewer-Specific Load Methods

Odon warm loads were triggered through the Odon MCP by calling the appropriate open-image control for the OME-Zarr path, then setting all channels visible and applying the warm-load contrast scale.

napari warm loads were triggered from Python by loading the OME-Zarr into an already-running napari process and making all layers visible.

QuPath warm loads used generated Groovy scripts. `make_qupath_warm_load_scripts.py` wrote all-channel QuPath image-loading scripts for each cell-count condition, and these scripts were executed in QuPath.

9.5 Timing Calculation

Load times were manually measured in Adobe Premiere from the visible load-start marker to the first frame where the image was considered viewable. Timecodes were stored as `MM:SS:FF` at 60 fps. The conversion was:

$$t_{\text{load}} = 60\,\text{minutes} + \text{seconds} + \frac{\text{frames}}{\text{fps}}$$

The warm-load analysis script grouped the three replicates by viewer, dataset cell count, file format, and visible channel count, then calculated:

$$\bar{t} = \text{mean}(t_{\text{load}})$$

$$s_t = \text{sd}_{sample}(t_{\text{load}})$$

$$\text{SEM}_t = \frac{s_t}{\sqrt{n}}$$

$$\text{CV}_{\%} = 100\frac{s_t}{\bar{t}}$$

The script also calculated a relative value versus Odon for the same cell count:

$$\text{relativeToOdon} = \frac{\bar{t}_{\text{viewer}}}{\bar{t}_{\text{Odon, same cell count}}}$$

9.6 Relevant Scripts and Outputs

All warm-start load benchmark scripts are archived under `benchmark_scripts/04_warm_start_load/`:

- `00_04_warm_start_load_file_descriptions.md`: folder-local inventory describing the warm-start load benchmark files.
- `00_run_warm_load.sh`: top-level warm-start load benchmark command sequence.
- `01_screen_marker.py`: screen-visible load marker used for video-timed warm-start benchmarking.
- `02_warm_load_common.py`: shared helpers for warm-start load scripts.
- `03_odon_warm_load_time.py`: Odon warm-start image-load driver.
- `04_napari_warm_load_time.py`: napari warm-start image-load driver.
- `05_make_qupath_warm_load_scripts.py`: generator for QuPath warm-start image-loading Groovy scripts.
- `06_01_qupath_warm_load_10k_channels_all.groovy`: QuPath 10k all-channel warm-start image-loading script.
- `06_02_qupath_warm_load_50k_channels_all.groovy`: QuPath 50k all-channel warm-start image-loading script.
- `06_03_qupath_warm_load_100k_channels_all.groovy`: QuPath 100k all-channel warm-start image-loading script.
- `06_04_qupath_warm_load_500k_channels_all.groovy`: QuPath 500k all-channel warm-start image-loading script.
- `06_05_qupath_warm_load_1m_channels_all.groovy`: QuPath 1m all-channel warm-start image-loading script.
- `07_analyze_warm_start_load_times.py`: manual warm-start load-time replicate analysis.

- `napari_scriptable_zoom.py`: import-compatible napari helper used by the warm-load driver.
- `odon_scriptable_zoom.py`: import-compatible Odon MCP helper used by the warm-load driver.
- `timecode_common.py`: shared Premiere MM:SS:FF timecode parsing helper.

Main outputs:

- `data/raw_replicates/warm_start_load_times_replicates.csv`
- `data/warm_start_load_times_summary.csv`

10 Benchmark 4: Level-0 Tile Load After Fast Zoom

10.1 Aim

This benchmark measures how quickly a viewer resolves full-resolution viewport tiles after a deliberately rapid zoom into the 1m-cell image. It complements the zoom-step error benchmark because a viewer can animate with a low motion residual while still failing to load high-resolution tile content in time.

10.2 Procedure

The benchmark used a zoom-in-only sequence:

- dataset: 1m cells;
- zoom factor: 1000;
- zoom duration: 0.1 s;
- file formats: OME-Zarr and OME-TIFF;
- viewers included in the final comparison: Odon and QuPath.

napari was not included in the final level-0 OME-TIFF comparison because direct loading of the large OME-TIFF was not stable during exploratory testing, and napari's multiscale behaviour during the rapid zoom did not produce the same visible endpoint as Odon and QuPath.

10.3 Endpoint Definition

The measured endpoint was the first video frame where level-0 viewport tiles visibly resolved after the fast zoom. Timing was performed manually in Adobe Premiere from the fast-zoom trigger to the visible level-0 tile-resolution frame.

The raw Premiere values were recorded as `MM:SS:FF` at 60 fps and converted as:

$$t_{\text{tile}} = 60\,\text{minutes} + \text{seconds} + \frac{\text{frames}}{\text{fps}}$$

For each `(viewer, file_format)` condition, three replicate values were summarized as:

$$\bar{t}_{\text{tile}} = \text{mean}(t_{\text{tile}})$$

$$s_{\text{tile}} = \text{sd}_{sample}(t_{\text{tile}})$$

$$t_{\text{min}} = \text{min}(t_{\text{tile}})$$

$$t_{\text{max}} = \text{max}(t_{\text{tile}})$$

The corrected summary means used for the final figure are:

- Odon OME-Zarr: 0.239 s;
- Odon OME-TIFF: 0.878 s;
- QuPath OME-Zarr: 3.500 s;
- QuPath OME-TIFF: 3.411 s.

10.4 Relevant Scripts and Outputs

All level-0 tile-load benchmark scripts are archived under `benchmark_scripts/05_level0_tile_load/`:

- `00_05_level0_tile_load_file_descriptions.md`: folder-local inventory describing the level-0 tile-load benchmark files.
- `00_run_level0_tile_load.sh`: focused command reference for the final 1m fast zoom-in level-0 tile-load benchmark runs.
- `01_odon_scriptable_zoom.py`: Odon fast zoom-in driver for the level-0 tile-load benchmark.
- `02_01_qupath_scriptable_zoom_in_only_1m_zarr_fast_channels_1.groovy`: QuPath 1m OME-Zarr fast zoom-in-only benchmark.
- `02_02_qupath_scriptable_zoom_in_only_1m_tiff_fast_channels_1.groovy`: QuPath 1m OME-TIFF fast zoom-in-only benchmark.
- `04_analyze_level0_tile_load_times.py`: fast level-0 tile-load timing analysis helper.
- `timecode_common.py`: shared Premiere MM:SS:FF timecode parsing helper.

Main outputs:

- `data/raw_replicates/level0_tile_load_times_1m_replicates.csv`
- `data/level0_tile_load_times_1m_summary.csv`

11 Figure Generation and Data Aggregation

Publication CSV files were generated from the benchmark analysis outputs with:

- `scripts/prepare_publication_benchmark_data.R`;
- archived bundle copy: `benchmark_scripts/06_publication_outputs/00_prepare_publication_benchmark_data.R`.

This script reads the analysis outputs, normalizes viewer and cell-count labels, converts memory from MB to GB for plotting, carries through replicate-level tables, and writes the curated files in `data/`.

The manuscript-style figure was generated with:

- `scripts/plot_manuscript_benchmarks.R`;
- archived bundle copy: `benchmark_scripts/06_publication_outputs/01_plot_manuscript_benchmarks.R`.

The plotting script uses `ggplot2` and `patchwork`. It applies the same viewer colour scheme across panels:

- Odon: `#EC2309`;
- napari: `#3943B7`;
- QuPath: `#F9DB6D`.

Bars show condition means. Points overlaid on bars show the three replicate measurements. Error bars represent the replicate standard deviation. QuPath zoom-step error conditions where level-0 tiles did not visibly resolve are shown as grey hatched bars.

12 Supplementary Media

The supplementary media folder contains selected short clips and transform previews rather than the full raw screen recordings. This keeps the distribution size manageable while preserving material that allows visual auditing of the conclusions.

Included media:

- side-by-side composite clips comparing Odon, napari, and QuPath for representative 1-channel zoom runs;
- individual 640 x 360 downsampled zoom clips;
- affine transform overlay images showing representative estimated frame-to-frame transforms;
- a supplementary media manifest.

The media subset was prepared with:

- `scripts/prepare_supplementary_assets.R`;
- archived bundle copy: `03_prepare_supplementary_assets.R` in the `06_publication_outputs` script-bundle folder.

The publication-output bundle folder also includes:

- `00_06_publication_outputs_file_descriptions.md`: folder-local inventory describing the publication-output scripts.
- `02_plot_affine_zoom_trace.R`: affine zoom-step trace and residual plot generator.
- `04_build_supplementary_methods_pdf.py`: supplementary methods PDF generator.
- `05_prepare_benchmark_script_bundle.py`: curated benchmark script bundle generator.

13 Interpretation and Limitations

These benchmarks support a specific visualization claim: Odon is lightweight and responsive for browsing large highly multiplexed OME-Zarr spatial proteomics data. The benchmarks do not claim that Odon replaces the broader analysis functionality of QuPath or napari. QuPath and napari support many analysis workflows and file-format scenarios beyond the scope of this focused visualization comparison.

The benchmark values depend on local machine state, viewer versions, screen recording settings, installed codecs, and the generated datasets. The scripts, curated data tables, and manifest are provided to make the methodology auditable and reproducible in principle, but paths and viewer installation locations must be adapted for another environment.

The P95 absolute zoom-step error measures visible frame-to-frame zoom-step residual, not biological image quality or tile-resolution success. Tile-resolution failures are therefore marked separately in the figure. Similarly, warm-start load time measures the first viewable state, not necessarily completion of all possible background tile loading.

14 Key Output Files

- `data/`: curated summary CSV files used in the manuscript figure.
- `data/raw_replicates/`: replicate-level data for each benchmark.
- `figures/`: final manuscript-style benchmark figure as PDF and PNG.
- `benchmark_scripts/`: final benchmark script bundle.

- `benchmark_scripts/script_manifest.csv`: manifest for bundled scripts.
- `benchmark_scripts/*/00_*_file_descriptions.md`: folder-local file inventories for each benchmark workflow.
- `supplementary/`: supporting clips and affine transform previews.
